# Complexities in Managing Psychosis in a Patient With Stiff-Person Syndrome: A Case Report

**DOI:** 10.7759/cureus.52930

**Published:** 2024-01-25

**Authors:** Marie Jean, Ramprasad Paidi, Gokul Paidi, Sayma Akhter

**Affiliations:** 1 Department of Psychiatry, Garnet Health Medical Center, Middletown, USA; 2 Internal Medicine, S. Nijalingappa Medical College, Bangalore, IND; 3 Internal Medicine, Abrazo Community Health Network, Glendale, USA; 4 School of Medicine, Touro College of Osteopathic Medicine, Middletown, USA

**Keywords:** psychiatric disorders, neurotransmitter, psychosis, muscle spasms, stiff person syndrome

## Abstract

Stiff-person syndrome (SPS) is an uncommon autoimmune neurological disorder marked by painful muscle stiffness, muscle spasms, and limb weakness. Neurological symptoms in SPS can mimic a psychogenic movement disorder in which symptoms are triggered by sudden movement and emotional distress, which might delay proper treatment. However, psychiatric symptoms are far less common, and there is limited understanding regarding the co-occurrence of psychiatric conditions. Psychiatric symptoms include nonspecific anxiety, agoraphobia, and depression, which can be triggered by sudden movement, noise, or emotional stress. This case report dives into the psychiatric manifestations seen in a patient with SPS. The case report focuses on a 42-year-old female with SPS, migraines, systemic lupus erythematosus, Sjogren's syndrome, and a psychiatric history of anorexia, depression, and anxiety. Her unique presentation underscored the necessity for a multidisciplinary approach to psychiatric care. The patient was evaluated and managed during her admission to the psychiatric unit for unspecified psychosis. Her course included a complicated medical evaluation for cardiovascular and neurologic symptoms and comprehensive psychiatric management. She manifested resistance to specific psychiatric medications and care strategies. She had atypical presentations, like sensory symptoms and left-sided chest pain. She exhibited paranoia and psychosis, which were managed with a combination of pharmacologic treatments, including aripiprazole. Psychotic symptoms were resolved upon discharge, with an emphasis on strict outpatient follow-up. This case report enhances our understanding of the clinical nuances associated with SPS and its intersection with psychiatric symptoms. The objective of this case report is to detail the diagnostic and therapeutic complexities of managing psychosis in a patient with SPS, along with a pre-existing complex medical and psychiatric profile, and to contribute to a deeper understanding of SPS and associated psychiatric conditions and more effective management strategies.

## Introduction

Stiff-person syndrome (SPS) is an uncommon autoimmune neurological disorder marked by painful stiffness and muscle spasms. Individuals with SPS might exhibit psychiatric symptoms, and there is limited understanding regarding the co-occurrence of psychiatric conditions. The condition referred to as SPS, alternatively known as stiff-man syndrome, was initially documented in 1956 [1]. It is more commonly observed in women, occurring two to three times more often than in men, and its prevalence is estimated to be around one in every one million people [2]. This is a rare neurological condition marked by muscle stiffness, rigidity, and muscle spasms, which can elevate the risk of falls for affected individuals [1,2]. There is a growing body of evidence supporting the idea that SPS is primarily an autoimmune disorder with a strong involvement in the central nervous system, stemming from B-cell-mediated production of autoantibodies targeting specific inhibitory regions on the enzyme glutamic acid decarboxylase (GAD) and the synaptic membrane protein amphiphysin [3].

Three primary forms of SPS are recognized based on their underlying pathophysiological mechanisms: autoimmune, paraneoplastic, and idiopathic SPS. In the autoimmune variant of SPS, the antibodies are targeted against a specific enzyme known as glutamic acid decarboxylase (GAD) [2]. In the paraneoplastic form, the antibodies may be directed at presynaptic proteins like amphiphysin or postsynaptic proteins like gephyrin [2,3].

The diagnosis of SPS should be established through a combination of clinical evaluation, laboratory tests, and electromyoneurographic assessments, following the criteria set forth by Gordon and Lorish [4]. Treatment strategies for SPS primarily revolve around symptomatic management to alleviate muscle spasms and may also include immunomodulatory interventions aimed at reducing the autoimmune response.

Alongside neurological abnormalities, individuals with SPS often receive diagnoses of psychiatric conditions like depression, anxiety, and phobias [5]. However, the specific management of psychosis in the context of SPS remains an underexplored area. Correctly identifying the diagnostic criteria and therapeutic interventions for psychosis in patients with SPS is essential to improving our understanding of the full clinical picture of the disease, thereby finding more targeted and effective approaches to treating patients.

## Case presentation

We present the case of a 42-year-old Caucasian female with a past medical history of SPS, migraines, systemic lupus erythematosus (SLE), Sjogren's syndrome, and a past psychiatric history of anorexia, depression, and anxiety. There was no history of suicide attempts. Her social history revealed she is a divorced woman with a master’s degree level of education who lives alone, separate from her children. In addition, social history revealed that the patient smokes marijuana two to three times a week.

**Figure 1:**
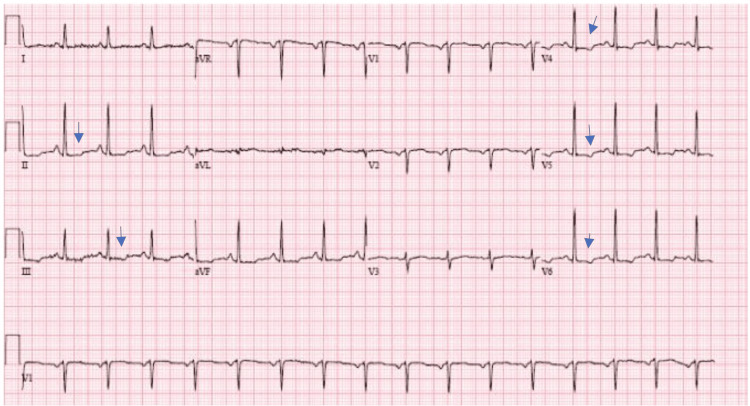
Electrocardiogram In the figure, the arrows point to areas of T-wave inversion

The patient presents with left-sided chest pain described as pressure that radiates to the left jaw and down the left arm with a 6/10 on the pain scale. The pain intensifies with deep breaths. She denies fever, chills, nausea, or vomiting. Additionally, she experiences numbness, paresthesias, and a presyncope feeling, with occasional incontinence during these episodes. The symptoms last for about an hour, and the patient reports a recurring sensation of feeling "like I am going to die." Vital signs on presentation were within normal limits, D-dimer was elevated at 1104, computed tomography (CT) angiography was negative for pulmonary embolism or dissection, and troponin I and creatine kinase were normal. A urine toxicology screen was positive for benzodiazepines and cannabinoids. Electrocardiogram testing showed T-wave inversions in the inferior and lateral leads (Figure 1).

Cardiovascular etiologies, including acute coronary syndrome, were ruled out with a normal echocardiogram and computerized tomography coronary angiogram. However, the patient continued to complain of intermittent chest pain. She subsequently developed left-sided paresthesia and presyncope, leading to a neurologic evaluation that returned negative results for electroencephalogram (EEG), magnetic resonance imaging (MRI), and normal carotid studies. The patient was continued on the home medication duloxetine, clonazepam, and intravenous immune globulin (IVIG) for SPS. She was provided with, prior to admission, trazodone for anxiety and insomnia, tramadol for pain, and topiramate for migraines. Amphetamine/dextroamphetamine was held. The patient's list of home medications is detailed in Table 1.

**Table 1:** Medication list: prior to admission ADHD: attention-deficit/hyperactivity disorder; SPS: stiff-person syndrome

Medication	Dose	Reason/Illness
Amphetamine-dextroamphetamine (Adderall XR)	15 mg (24 hr cap)	ADHD, unspecified ADHD type (held)
Duloxetine (Cymbalta)	30 mg	SPS
Immune globulin, human (Gammunex-C 10%)	60 G (inject)	SPS
Rituximab-Abbs (Truxima)	1,000 mg (inject)	SPS
Topiramate (Topamax)	200 mg	Migraines
Clonazepam (Klonopin)	2 mg (morning), 2 mg (evening)	SPS
Tramadol (Ultram)	50 mg (Q6H PRN)	Pain
Trazodone (Desyrel)	50 mg	Insomnia and anxiety

Her neurological symptoms improved. But on day three of hospitalization, her hospital course was complicated by the onset of delusions, paranoia, and auditory hallucinations, necessitating psychiatric evaluation and treatment. She was diagnosed with psychosis of an unspecified type. She demonstrated resistance to the proposed psychiatric care plan, which consisted of continuing trazodone, starting aripiprazole for psychosis, and having a one-on-one sitter for constant observation. The patient selectively declined aripiprazole while consenting to her other medications.

After internal medicine clearance, her psychosis, danger to herself, inability to care for herself due to mental illness, and the need for psychotropic medication stabilization necessitated a transfer to the behavioral health unit (BHU). The mental status examination revealed delusional and paranoid thought content, accompanied by auditory hallucinations, as detailed in Table 2.

**Table 2:** Mental status evaluation MSK: musculoskeletal

Mental status evaluation details	
Appearance	Appears stated age
MSK/gait	Unsteady, uses a walker
Behavior	Uncooperative
Language	Fluent in English
Speech	Regular rate and regular volume
Mood	“Frustrated”
Affect	Mood-congruent
Thought process	Disorganized, confused
Thought content	Delusions, paranoia, denies suicidal/homicidal ideation, intent, or plan,
Preception	Auditory hallucinations elicited
Attention/concentration	Grossly intact
Sensorium	Person, place, and time/date
Memory	Grossly intact
Fund of knowledge	Average
Cognition	Grossly intact
Insight	Fair
Judgment	Fair

In the BHU, she remained uncooperative, refrained from group therapies, and continued to display signs of psychosis. Six days later, she was readmitted to the medical floor for new-onset left facial numbness and otalgia accompanied by tinnitus, of unclear etiology. She was subsequently treated for a urinary tract infection (UTI), following positive leukocytes detected in her urinalysis results. Her headache management was escalated with valproate 250 mg BID, along with the continuation of topiramate. 

Throughout her second inpatient stay on the medical unit, the patient received regular IVIG treatments for SPS, completing a dose of 60 grams. Despite extensive workup, which included repeat CT brain, B12 and folate levels, and Lyme antibodies, her neurological symptoms were deemed non-specific and resolved. She was transferred, about one week later, back to the BHU for continued psychiatric care due to ongoing psychosis.

Upon re-evaluation at the BHU, the patient appeared calm, oriented and engaged. She denied any current suicidal or homicidal ideation, intent, or plan and reported no hallucinations, paranoia, or delusions at the time of examination. However, the patient exhibited a paranoid thought process and minimized her behaviors.

A psychiatric review of the systems revealed intermittent episodes of anxiety and low mood without the full spectrum of depressive symptoms. She did not report any manic symptoms at the time of the evaluation. Her diagnosis on admission was unspecified psychosis. The differential diagnoses included major depressive disorder (MDD), single episode, severe, with psychosis, and cannabis-induced psychosis. MDD, single episode, severe, with psychosis, was ruled out due to a lack of depressive symptoms. While cannabis-induced psychosis, although not likely, due to the patient's psychotic symptoms being present for about one month in the absence of marijuana use, could not be conclusively ruled out.

A comprehensive review of the patient's medical record demonstrated that the patient has a history of manipulative behaviors, such as feigning ignorance and diverting topics, coupled with a guarded attitude regarding her paranoid delusions. Additionally, she faces multiple psychosocial stressors, including an ongoing custody battle and an order of protection from her ex-husband. Despite contracting for safety and agreeing to comply with treatment, during admission, collateral reports raised significant concerns for her safety, believing that the patient was a danger to herself.

Although the patient had no psychiatric history of psychosis and was not on any antipsychotics prior to this hospital course, collateral information indicated that the patient's delusions sometimes led her to engage in unsafe behavior, such as dismantling light fixtures and inserting a tweezer inside to search for hidden cameras that she believes are being used to spy on her. Due to collateral accounts and recent unsafe behaviors observed on the medical floor, such as the patient leaving her room to search for family members outside visiting hours, a precautionary measure of 15-minute safety room checks was implemented for the patient.

The treatment plan involved the continuation of aripiprazole 5 mg, which the patient agreed to after initial refusal, management of anxiety and insomnia with trazodone 50 mg, ongoing treatment of SPS with IVIG, duloxetine 30 mg, and clonazepam 2 mg, and treatment of migraines with topiramate 200 mg. Her UTI was addressed with a course of levofloxacin 500 mg.

**Figure 2:**
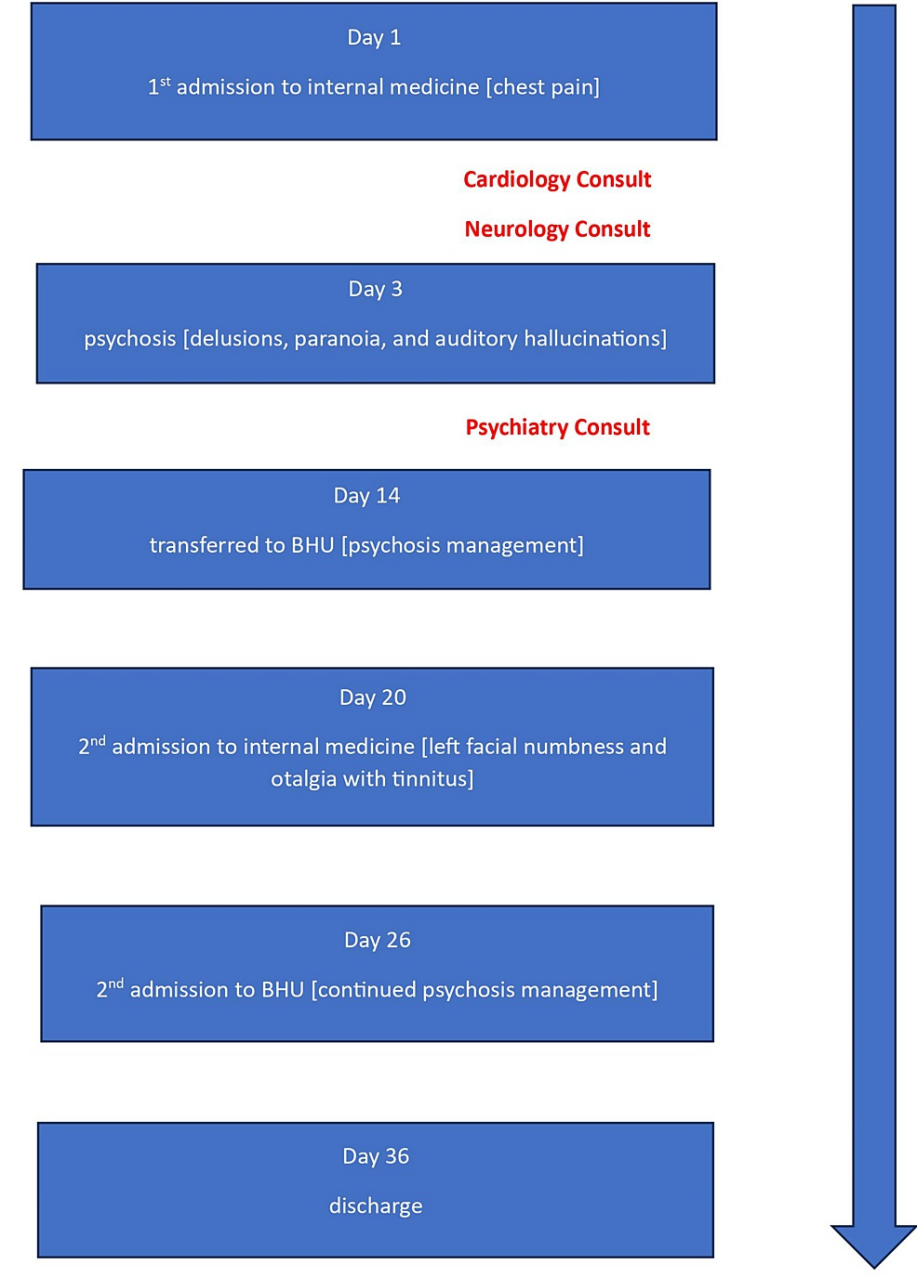
Hospital timeline BHU: behavioral health unit

Subsequently, after ten days of in-patient treatment, she improved. The patient was discharged home in stable condition with resolved psychosis, as outlined in Figure 2. Outpatient follow-up and medication adherence were emphasized in the discharge treatment plan.

## Discussion

**Figure 3:**
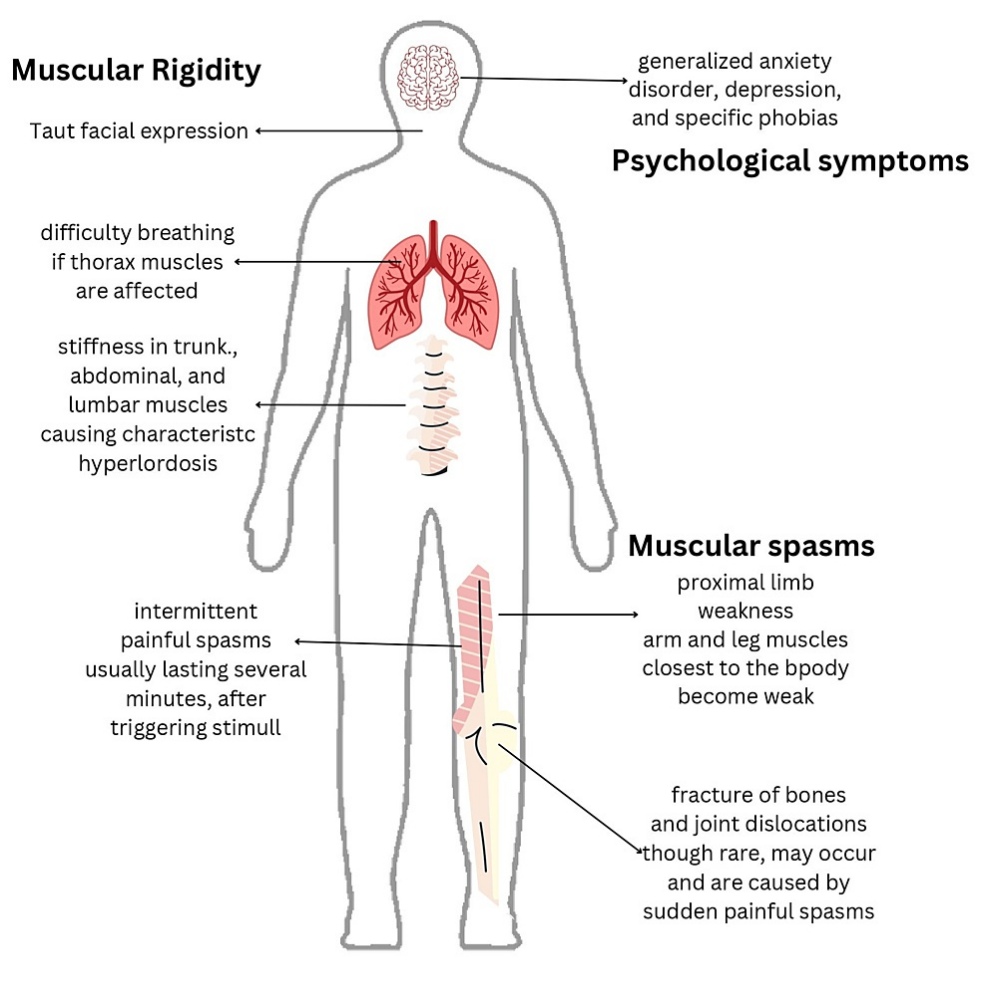
Symptoms of SPS (stiff-person syndrome) Image credits: Gokul Padi, Marie Jean

The exact mechanism of action responsible for psychiatric symptoms in SPS is not fully understood. It is believed to involve a complex interplay of factors, such as neurotransmitter imbalance, autoimmunity, brainstem involvement, and the psychological effects of chronic illness [5]. It is associated with dysfunction in the inhibitory neurotransmitter system, particularly involving gamma-aminobutyric acid (GABA) [6]. Low levels of GABA may contribute to both the motor symptoms and psychiatric manifestations seen in SPS [6]. Evidence suggests an autoimmune component in SPS, where the immune system mistakenly targets the body's own cells, including those in the central nervous system [5,6]. Autoimmune processes may contribute to the neurological and psychiatric symptoms observed in SPS [2]. Some research indicates that abnormalities in the brainstem may play a role in SPS [2]. Dysfunction in this area could contribute to both motor and psychiatric symptoms [2]. Also, dealing with a chronic and often disabling condition like SPS can lead to psychological distress, including anxiety and depression [5]. In concurrence, our patient has a history of anxiety and has displayed both psychotic features and symptoms associated with SPS, including stiffness in the trunk, limb weakness, and facial numbness simultaneously. Figure 3 highlights the symptoms of SPS.

A case study involving a 51-year-old woman with advanced SPS and generalized anxiety disorder provided evidence that benzodiazepines, which boost GABAergic inhibition, are beneficial [6,7]. The patient's increased use of clonazepam, as in our case, was associated with reduced stiffness and anxiety, suggesting effectiveness despite potential confounding variables [6]. In our case, the patient was medically cleared by neurology and cardiology. Yet, her symptoms usually persisted for some time after medical clearance. Often, the lack of neuropathological findings is markedly at odds with the profound disability seen in those with SPS, implying that the dysfunction of inhibitory circuits, even without anatomical damage, is adequate to produce the complete range of clinical manifestations seen in SPS [3]. This can lead to misdiagnoses. Sabatino et al. (2016) report on five individuals with SPS who were initially misdiagnosed with multiple sclerosis (MS) [7]. This includes a case where the patient received three distinct MS treatments due to what was perceived as 'disease progression' [7]. These instances underscore the importance of considering rarer neuroimmunological conditions like SPS, particularly in patients exhibiting unusual characteristics for MS. Likewise, the Souissi et al. (2020) case study highlights the diagnostic challenge of SPS diagnosis. Souissi et al. (2020) explored the case of a 36-year-old man whose 11-year neuromuscular deterioration was originally misdiagnosed as psychiatric in nature [8]. His symptoms were characterized by persistent muscle contractions and pronounced adrenergic responses, which were particularly striking given the normal imaging and electrophysiological results. However, elevated anti-GAD65 antibodies confirmed an autoimmune etiology for his SPS. The successful treatment with pregabalin underlines the importance of immunological markers for accurate diagnosis and the efficacy of anticonvulsants in the therapeutic approach [8,9]. This case stresses the imperative of clinical awareness for atypical neuroimmunological conditions and illuminates the presumed autoimmune mechanism of SPS, which is complicated by its rarity and the lack of standardized treatment protocols. Similar to our case, Sanak et al. presented a case that detailed a patient with SPS and psychosis who improved with Depakote and risperidone [9]. However, there is limited literature available on the association between SPS and psychosis, highlighting a gap in knowledge in this area.

While scarce, research has demonstrated that it is critical to address psychiatric symptoms in patients with SPS. Patel et al. (2020) described a multi-comorbid 58-year-old female patient with SPS who experienced exacerbations of her symptoms due to an unmanaged panic disorder [10]. Despite using various medications for her bipolar and panic disorders, diabetes, and hypothyroidism, she still faced significant stiffness and tremors. Benzodiazepine therapy was found to be most effective for her SPS symptoms, while selective serotonin reuptake inhibitors (SSRIs) were inadequate. This case stresses the potential of benzodiazepines in SPS psychiatric symptom management and calls for more research to fine-tune treatments for such intricate medical profiles [9,10]. Continuous medication management and the role of a clinical pharmacist were emphasized as essential to patient care.

Building on this imperative, Caffrey et al.'s (2021) systematic review of 27 studies indicated that SPS patients have a higher risk of psychiatric conditions such as generalized anxiety disorder, substance abuse, major depressive disorder, and unspecified psychiatric disorders than the general public, with risks comparable to those with multiple sclerosis. However, small sample sizes led to broad confidence intervals [11]. The study also highlighted that SPS diagnoses are frequently delayed by the misinterpretation of symptoms as purely psychiatric. The findings underscore the importance of psychiatric evaluation in the timely diagnosis and treatment of SPS, suggesting that psychiatric symptoms should expedite rather than hinder the diagnosis of SPS. The Nasri et al. study also emphasized the necessity of early detection of psychiatric symptoms to facilitate prompt SPS diagnosis and improve patient outcomes. Nasri et al.'s (2023) review reported on two SPS cases with phobic disorder and analyzed 239 patients with SPS-related psychiatric symptoms, noting anxiety and depression as the most common [12]. They observed that the classic form of SPS was predominant and that autoimmune factors were the main cause.

The challenge of accurately diagnosing SPS in the presence of psychiatric illness is further complicated by treatment decisions. The uncertainties surrounding the role of certain medications in SPS are highlighted by Culav-Sumić et al. (2008) and Benavides et al. (2016). Culav-Sumić et al. (2008) described a patient with SPS and depression who did not respond to treatment with paroxetine or high-dose diazepam, despite an adequate trial period and dosing, resulting in persistent anxiety and depression symptoms [13]. Benavides et al. (2016) reported on four cases demonstrating that the initiation of serotonin-norepinephrine reuptake inhibitors (SNRI) aggravated SPS symptoms, such as increased spasms and cognitive haze, despite using adjunctive benzodiazepines and intrathecal baclofen [14,15]. Given the lack of guidelines for SSRI or SNRI use in SPS and mixed evidence, the role of these medications remains unclear, amplifying the need for further research [13]. However, benzodiazepines, particularly clonazepam, in managing both motor and psychiatric symptoms in SPS are more promising.

## Conclusions

This case illustrates the complexity of managing a patient with stiff-person syndrome and psychosis with a multi-faceted medical and psychiatric presentation. The combined presentations of chronic autoimmune disorders, acute medical issues, and psychiatric symptoms pose a challenge for clinicians, highlighting the necessity of a comprehensive, multidisciplinary approach to care. Also, this case highlights the importance of developing standardized guidelines for the use of psychiatric medications in SPS, considering the potential exacerbation of symptoms observed in some instances. In addition, current evidence suggests that a relationship may exist between SPS and psychiatric disorders. The proposed connection between the two requires further research to enhance the medical management of patients with SPS and psychiatric illnesses.
